# Molecular tiling on the surface of a bacterial spore – the exosporium of the *Bacillus anthracis/cereus/thuringiensis* group

**DOI:** 10.1111/mmi.13650

**Published:** 2017-03-08

**Authors:** Cassandra Terry, Shuo Jiang, David S. Radford, Qiang Wan, Svetomir Tzokov, Anne Moir, Per A. Bullough

**Affiliations:** ^1^ Krebs Institute for Biomolecular Research, Department of Molecular Biology and BiotechnologyUniversity of SheffieldSheffieldUK; ^2^ Present address: MRC Prion Unit and Department of Neurodegenerative DiseaseUCL Institute of NeurologyLondonUK; ^3^ Present address: Department of Molecular Biology, Massachusetts General Hospital, and Department of GeneticsHarvard Medical SchoolBostonMAUSA; ^4^ Present address: School of Biological SciencesUniversity of EdinburghEdinburghUK.

## Abstract

Bacteria of the genera *Bacillus* and *Clostridium* form highly resistant spores, which in the case of some pathogens act as the infectious agents. An exosporium forms the outermost layer of some spores; it plays roles in protection, adhesion, dissemination, host targeting in pathogens and germination control. The exosporium of the *Bacillus cereus* group, including the anthrax pathogen, contains a 2D‐crystalline basal layer, overlaid by a hairy nap. BclA and related proteins form the hairy nap, and require ExsFA (BxpB) for their localization on the basal layer. Until now, the identity of the main structural protein components of the basal layer was unknown. We demonstrate here that ExsY forms one of the essential components. Through heterologous expression in *Escherichia coli*, we also demonstrate that ExsY can self‐assemble into ordered 2D arrays that mimic the structure of the exosporium basal layer. Self‐assembly is likely to play an important role in the construction of the exosporium. The ExsY array is stable to heat and chemical denaturants, forming a robust layer that would contribute to overall spore resistance. Our structural analysis also provides novel insight into the location of other molecular components anchored onto the exosporium, such as BclA and ExsFA.

## Introduction

Bacterial endospores are differentiated cell forms that are specialized to survive extreme environmental stress (Setlow, 2014); in some pathogens, such as *Bacillus anthracis* and *Clostridium difficile*, the spore acts as the infectious agent. Spores constitute a fascinating system for exploration of fundamental principles in cell development and assembly of complex supramolecular structures (Henriques and Moran, 2007; Giorno *et al*., 2007; Mckenney *et al*., 2013; Jiang *et al*., 2015). Following an asymmetric cell division the forespore is engulfed by the larger mother cell; subsequently, cortex peptidoglycan and protein coat layers are laid down, surrounding the cellular core in which the genome is protected. Once assembly is complete, the mature spore is released from the mother cell. The protein coat confers much of the spore's resilience and resistance to biocidal chemicals and enzymes (Henriques and Moran, 2007; Setlow, 2014).

Spores of some species including *Bacillus anthracis* have an exosporium (Henriques and Moran, 2007; Stewart, 2015) that is typically a flexible envelope surrounding the spore, with an ill‐defined interspace separating it from the protein coat. The exosporium defines the boundary between the spore and the environment (or host) with which it interacts. For pathogens, that interaction includes the first point of contact of the spore with cells of the host's immune system. The exosporium layer has multiple functions: it can resist chemical and enzymatic attack; it provides a surface for adhesion; it can harbour enzymes that modulate spore germination and may protect spores from macrophage‐mediated killing (Weaver *et al*., 2007; Stewart, 2015). Exosporium has been most extensively studied (reviewed in Stewart, 2015) in the *Bacillus cereus* group, which includes *B. anthracis* (causative agent of anthrax), *B. cereus* (a food‐borne pathogen) and *B. thuringiensis* (an insect pathogen). We restrict our attention to this *B. cereus sensu lato* group, in which the exosporium composition and structure are very similar (Ball *et al*., 2008); the exosporia of other organisms, such as *Bacillus megaterium* (Manetsberger *et al*., 2015), *Clostridium sporogenes* (Janganan *et al*., 2016) and *C. difficile* (Diaz‐Gonzalez *et al*., 2015) have very different protein compositions, although the general principles of assembly might have similarities.

Across species, the basic design of the exosporium consists of a thin continuous proteinaceous layer – the basal layer. Typically, some or all of the basal layer is crystalline (Gerhardt and Ribi, 1964; Ball *et al*., 2008; Stewart, 2015; Janganan *et al*., 2016). The external face of this layer may be embellished with filaments and appendages such as a ‘hairy nap’ (Gerhardt and Ribi, 1964; Driks, 2007). In the *B. cereus sensu lato* group the hairy nap is made of collagen‐like filaments (Sylvestre *et al*., 2003) predominantly composed of BclA, on the outermost surface of the basal layer. This BclA glycoprotein may have a role in spore protection (Boydston *et al*., 2005) and may play a role in host phagocytosis of the spore (Gu *et al*., 2012). It has recently been shown to mediate an immune inhibition mechanism that promotes spore persistence in mouse lung (Wang *et al*., 2016). ExsFA (also named BxpB) is a 17 kDa exosporium protein required for the efficient attachment of BclA to form the nap on the exosporium (Steichen *et al*., 2005; Sylvestre *et al*., 2005; Thompson *et al*., 2011). Enzymes sequestered to the exosporium, inosine hydrolase and alanine racemase (Todd *et al*., 2003; Redmond *et al*., 2004) may suppress premature germination by metabolizing potential germinants (Yan *et al*., 2007; Chesnokova *et al*., 2009).

The multifunctional exosporium has a number of structural demands placed on it (Ball *et al*., 2008; Kailas *et al*., 2011). It is semipermeable, breached by passages as narrow as ∼20 Å in diameter; these are small enough to exclude typical proteolytic enzymes but large enough to allow entry of spore germinants. The crystalline basal layer forms an array of interlinked cups opening to the external environment of the spore (Kailas *et al*., 2011). The cups could act as a matrix to sequester various additional proteins and enzymes associated with the exosporium. The exosporium is highly deformable, which may enhance the surface‐to‐surface contact area.

The exosporium contains a large number of proteins; however, until now we have understood little of the core proteins essential to the integrity of the exosporium and the way they assemble; for example, the structural protein(s) responsible for the crystalline basal layer have not been defined (Stewart, 2015). A major high‐molecular weight complex (> 200 kDa) that is not fully dissociated on SDS‐PAGE, is proposed to include BclA, ExsFA and also ExsY (Steichen *et al*., 2003; Redmond *et al*., 2004). The ExsY protein is a strong candidate for a major protein of the basal layer. It is certainly crucial for normal exosporium assembly; *B. cereus* (Johnson *et al*., 2006) and *B. anthracis* (Boydston *et al*., 2006) *exsY* mutant spores possess only a cap of exosporium at one pole. This residual exosporium cap contains a close homologue of ExsY, named CotY, which has ∼84% amino acid identity with ExsY with most of the differences in the extreme N‐terminal region. CotY protein is also present in the cap region of the wild type exosporium (Thompson *et al*., 2012). CotY is not essential for formation of an exosporium, however, as spores of a *cotY* mutant have an intact exosporium fully encircling the spore (Johnson *et al*., 2006). The *exsYcotY* double mutant spore has no exosporium (Johnson *et al*., 2006) and a very thin spore coat, with loose coat fragments in the preparation, demonstrating that these proteins have also a role in spore coat assembly. Formation of a cap at one pole is a distinctive morphological intermediate in exosporium assembly. The cap appears to be attached to a fragile sublayer, or sac, approximately the size of the exosporium (Boydston *et al*., 2006). Extension of the mature exosporium beyond the cap and around the developing spore requires ExsY (Boydston *et al*., 2006; Johnson *et al*., 2006; Thompson *et al*., 2012).

We previously demonstrated by electron microscopy (EM) that an orthologous protein CotY, forming part of the thick outer spore coat layer (the crust) in the non‐exosporium forming *B. subtilis* (and called CotY_Bs_ here for clarity) formed ordered stacks of 2D crystalline layers within an *Escherichia coli* expression host (Jiang *et al*., 2015). Individual layers were made up of a lattice of hexameric ring‐like particles, somewhat reminiscent of the exosporium basal layer of *B. cereus* (Ball *et al*., 2008). Given the ∼35% identity between CotY_Bs_ and *B. cereus* ExsY proteins, we speculated that ExsY might be key to forming the crystalline lattice within the exosporium (Jiang *et al*., 2015); we have investigated this here. However, until now, there has been no definitive and direct observation of an ExsY/CotY type of assembly in any native spore structure.

Our studies of coat proteins in *B. subtilis* suggest that self‐organization plays a very significant role in spore construction (Jiang *et al*., 2015). Does self‐assembly play a role in the extension of the exosporium around the nascent spore? What are the components and what are the interactions between these components of the exosporium responsible for such a robust yet thin, flexible and semi‐permeable structure? We attempt to address these questions in this paper.

## Results

### Strains studied

To identify maximally denaturing conditions, two strains were used: our well‐characterized laboratory *B. cereus* strain ATCC 10876 (referred to as *B. cereus*) and the nap‐less strain *B. thuringiensis kurstaki* 4D11, an acrystalliferous derivative of the HD1 strain (Stahly *et al*., 1978), referred to as *Bt* 4D11. Together these were previously used to identify the orientation of the basal layer relative to the exterior hairy nap (Kailas *et al*., 2011).

### Isolation and disassembly of exosporium crystals

**Figure 1:**
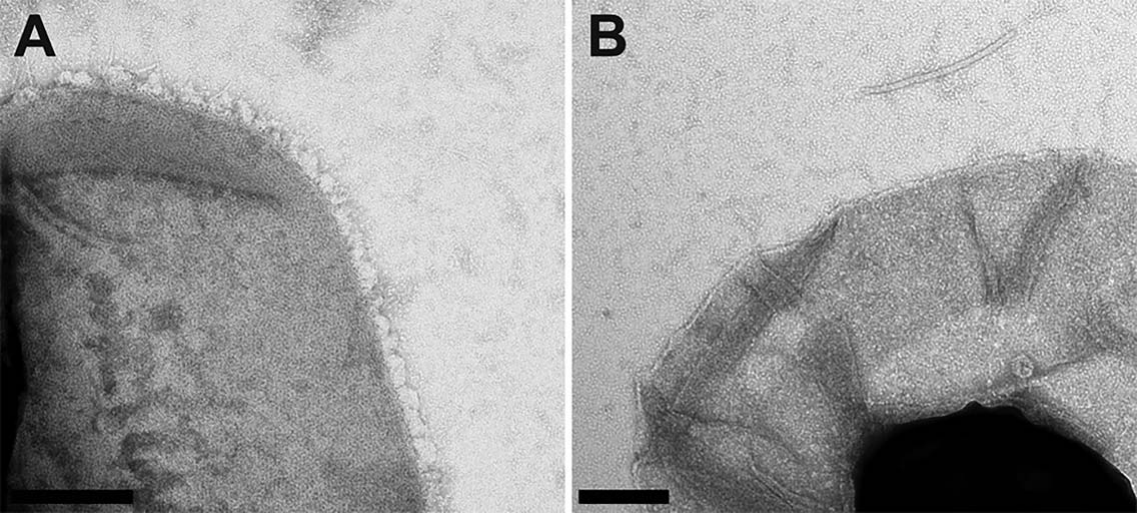
Peripheral regions of whole *Bacillus* spores examined by negative stain EM [Scale bars, 200 nm (A and B)]. A. *B. cereus* 10876. B. *B. thuringiensis* 4D11.

**Figure 2:**
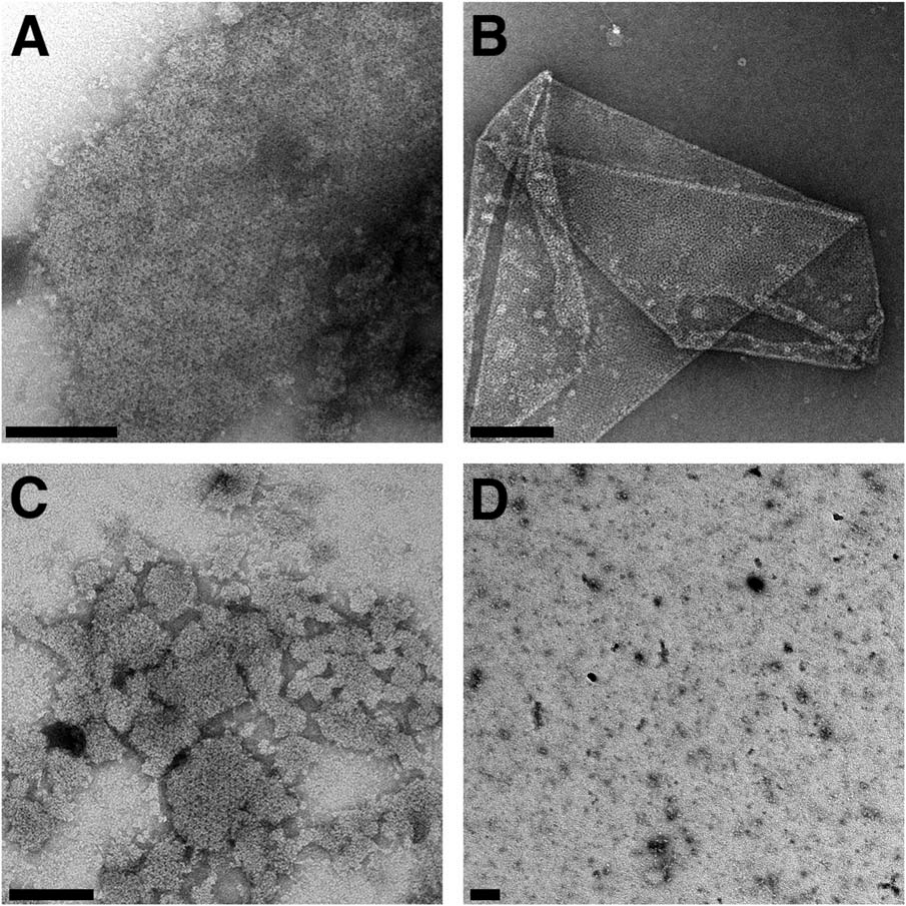
Negative stain EM of exosporium before and after disruption by chemical and heat treatment [Scale bars, 100 nm]. Fully washed exosporium crystals from (A) *B. cereus* 10876 and (B) *B. thuringiensis* 4D11. (C) *B. cereus* 10876 sample after treatment for 1 h with 1% SDS alone followed by dialysis of the soluble fraction. (D) After treatment of *B. cereus* 10876 exosporium crystals with 8 M urea, 2% SDS, 0.2 M DTT and incubating at 90°C.

EM of whole spores (Fig. 1) and of fully washed exosporium fragments (Fig. 2A and B) confirm the absence of nap in the *Bt* 4D11 strain. The images of the *B. cereus* crystal (Fig. 2A) presented less distinct lattice contrast and a peripheral fringe reflecting the presence of the hairy nap in this strain. Fragments from both strains were very robust to chemical treatment. With 1% SDS alone the soluble fraction, after centrifugation, yielded ‘spheroidal particles’, 10–45 nm in dimension and a few small crystalline fragments, all less than 80–90 nm on a side (Supplementary Information Fig. S1); the insoluble fraction still contained large intact exosporium fragments. After 72 h dialysis of the ‘soluble’ fraction, very few ‘spheroidal particles’ remained but crystalline fragments comparable to those seen in the starting material were visible (up to 300 nm on a side, Fig. 2C); these crystals displayed the same unit cell parameters as native exosporium. We observed similar dissociation/re‐association behaviour in the soluble fraction when 8 M urea was used instead of SDS.

Complete dissolution of exosporium crystals required incubation in 8 M urea, 200 mM DTT and 2% (w/v) SDS at 90°C for 20 min (‘harsh solubilization’ conditions); nevertheless, some particulate material remained in suspension (Fig. 2D). Upon dialysis large amorphous aggregates were formed but no crystals appeared.

### Maximizing the solubilization of exosporium fragments for identification of proteins

Many proteins have been identified as constituents of the exosporium, but it has been difficult to identify which of these are core building blocks essential to make up the repeating units of the crystalline basal layer (Ball *et al*., 2008; Kailas *et al*., 2011; Stewart, 2015). The insoluble fraction of exosporium that does not enter gels under standard conditions of SDS‐PAGE represents a major challenge in identifying the main structural components of the exosporium. In an attempt to identify the most abundant exosporium proteins common to both, we incubated extensively washed fragments of exosporium from *B. cereus* ATCC 10876 and *B. thuringiensis* 4D11 in urea, reducing agent, detergent and heat‐ ‘harsh solubilization’. This disruption of higher molecular weight complexes was reflected in the extensive dismantling of exosporium crystals, which we viewed by EM (Fig. 2D).

**Figure 3:**
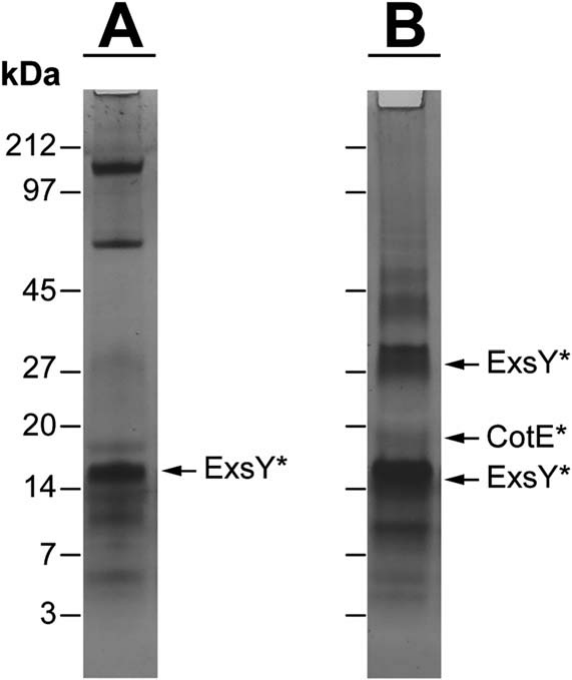
(A) *B. cereus* 10876 and (B) *B. thuringiensis* 4D11 exosporium fragments were isolated, washed and treated with 8M urea, 2% SDS, 0.2M DTT and heated to 90°C prior to SDS‐PAGE analysis. Bands corresponding to proteins identified by N‐terminal sequencing are marked by *.

**Figure 4:**
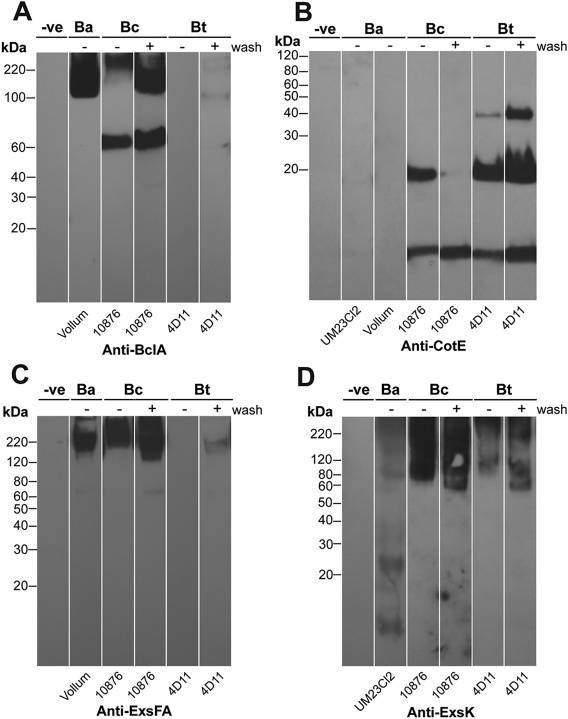
Differences in exosporium protein composition between *B. anthracis*, *B. cereus* and *B. thuringiensis* revealed by Western blotting. Exosporium fragments isolated from *B. anthracis* (Ba), *B. cereus* (Bc) and *B. thuringiensis* (Bt) spores were either unwashed (−) or fully washed (+) and analyzed using (A) anti‐BclA (B) anti‐CotE, (C) anti‐ExsFA or (D) anti‐ExsK antibodies. *B. cereus* 10876 vegetative cell extracts were used as a negative control (−ve). An equivalent amount of protein was added to each gel lane.

Proteins extracted from the fully washed crystals after ‘harsh solubilization’ treatment were analyzed by SDS‐PAGE. The exosporium samples dissociated sufficiently to reveal the predominance of a relatively small number of intensely staining, sharp, low molecular weight bands (Fig. 3). Unlike past protein profiles of our *B. cereus* strain, obtained under a more standard extraction procedure (Todd *et al*., 2003), the most abundant protein detected was now at 16kDa; this protein is also abundant in the *Bt* 4D11 strain, and was identified as ExsY through N‐terminal sequencing (SCNENKHHGS for *B. thuringiensis* 4D11 and SCNEN for *B. cereus*). A strong band at 32 kDa in *B. thuringiensis* was also identified as ExsY (SXNENXHH), corresponding in size to a likely dimer as only one sequence was detected. In *B. cereus* ATCC 10876 exosporium, sharp bands were visible at ca. 60 and 180 kDa along with a residual smear of larger material. These correspond to the position of BclA in Western blots (Fig. 4). Staining of any high molecular weight material above ∼50 kDa was very faint for the *Bt* 4D11 sample (Fig. 3B), as would be expected for an exosporium depleted in BclA‐containing hairy nap. CotE (SEFREIIT) was identified as a less prominent band at 20 kDa in the *Bt* 4D11 extract. We were unable to obtain N‐terminal sequences from the other, more minor bands at <50 kDa.

### Identification of additional proteins

The most abundant exosporium protein common to the two strains was identified above as ExsY (Fig. 3). Western blots probed more sensitively for the presence of additional candidate proteins from these and other *B. cereus sensu lato* strains (Fig. 4) using fully washed crystals after ‘harsh solubilization’ treatment. *B. anthracis* samples that had not undergone salt and SDS washes, available from previous experiments (Redmond *et al*., 2004; Ball *et al*., 2008) were included where available, as additional positive controls. BclA was detected in *B. cereus* and *B. anthracis* in a high molecular weight smeared band at ∼120 to 250 kDa, and in addition at ∼60 kDa in *B. cereus*. Very little was detected in the exosporium of *Bt* 4D11. ExsFA (predicted monomer 17 kDa) was detected in *B. cereus* and *B. anthracis* in the high molecular weight smear; again, only a trace was detected in *Bt* 4D11 exosporium. The very marked depletion of these two proteins is consistent with the absence of hairy nap in exosporium of this strain. Another exosporium protein ExsK (Redmond *et al*., 2004; Severson *et al*., 2009; predicted monomer 12 kDa) was detected in *B. cereus*, *B. anthracis* and *Bt* 4D11 in the high molecular weight smear, indicating that it, too is a component of a stable high molecular weight complex. Fainter bands at 12 and 25 kDa were also present in the *B. anthracis* lane, possibly corresponding to a monomer and dimer, as previously observed (Severson *et al*., 2009). CotE (predicted MW 20 kDa) was detected as a 20 or 8 kDa band, or both, in all *B. cereus* and *B. thuringiensis* strains tested but was not detected in *B. anthracis* exosporium, as already reported by Giorno *et al*. (2007). In *B. thuringiensis*, CotE was also detected as a ∼38 kDa band, probably a dimer. These data confirm the presence of CotE and ExsK common to the exosporium of *B. cereus* and *B. thuringiensis* 4D11. We can exclude CotE as a major component of the crystalline basal layer as the lattice observed for *B. anthracis* is the same as those of other *B. cereus sensu lato* strains (Ball *et al*., 2008); CotE is required for assembly rather than structure of the exosporium layer (Bressuire‐Isoard *et al*., 2016). Although we detected ExsK, *B. anthracis exsK* mutant spores were reported to have a normal intact exosporium (Severson *et al*., 2009), which excludes it as a strong candidate for a main crystalline protein. This leaves ExsY as a likely major component of the exosporium basal layer, on biochemical as well as previous genetic grounds.

### Heterologous expression of ExsY

**Figure 5:**
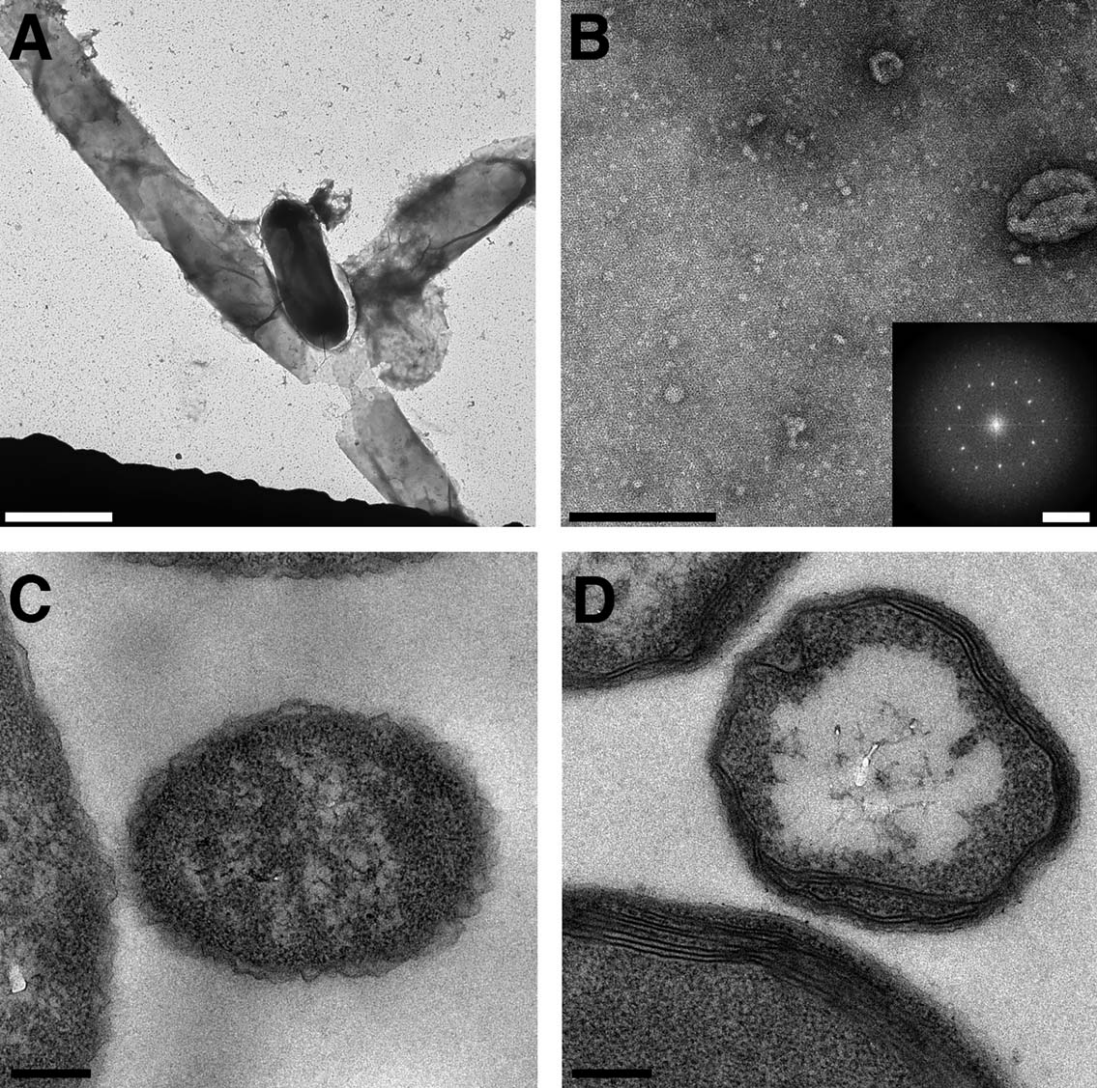
EM of 2His_6_‐ExsY crystals [Scale bars 2µm (A), 200 nm (B, C and D) and 0.28 nm^−1^ (B inset)]. A. rExsY crystals from lysed *E. coli* cells, along with a remaining intact cell and (B) smaller rExsY crystal fragment with inset displaying diffraction. Cross‐section through *E. coli* cell expressing (C) pET28a control vector and (D) rExsY.

The *exsY* gene from *B. cereus* was cloned and overexpressed in *E. coli*. *E. coli* cells expressing N‐ and C‐terminally poly‐histidine tagged ExsY were lysed by sonication in a urea‐containing buffer to release large, crystalline, proteinaceous sheets, (>1 μm on an edge) (Fig. 5A and B). These sheets were formed intracellularly (Fig. 5C and D). Cloned, untagged ExsY formed the same self‐assembled crystalline sheets, but for ease of purification, the double‐tagged construct (2His_6_‐ExsY) was used; we denote this recombinant form as rExsY. Crystals, affinity‐purified on NiNTA‐agarose beads were eluted using imidazole/urea, pelleted and resuspended in urea buffer; soluble rExsY remained in the supernatant. Recombinant *B. cereus* CotY also yielded crystalline arrays (Supplementary Information Fig. S2). However, these were less abundant and substantially more disordered than those of rExsY.

**Figure 6:**
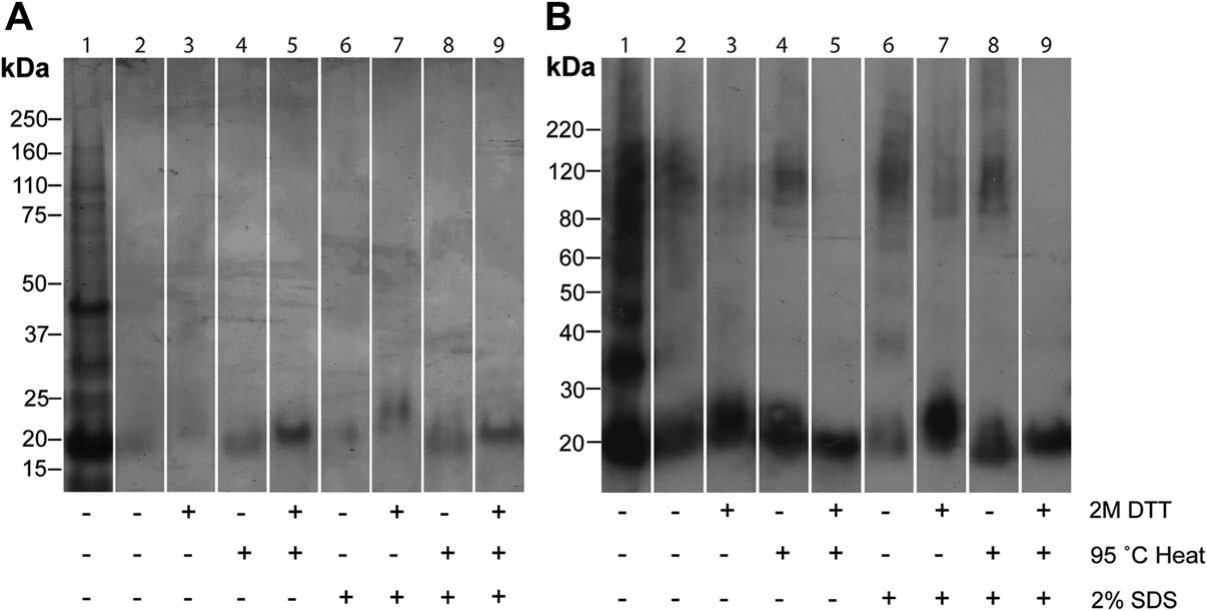
SDS‐PAGE analysis of rExsY crystals disrupted in 8 M urea using combinations of 2% SDS, 2 M DTT and 95°C. A. Stained with Coomassie blue and (B) after Western blotting with anti‐His_6_ antibody. Lane 1 corresponds to the soluble fraction whereas lanes 2–9 correspond to the rExsY crystals treated as indicated. An equivalent amount of protein was added to each gel lane with the exception of the soluble purified fraction.

The stability of rExsY crystals suspended in 8 M urea was tested in reducing, high temperature and detergent conditions (Fig. 6). As a control, the material from the soluble rExsY fraction in urea above was resolved on SDS‐PAGE gels into a ladder of proteins from ∼20 kDa upwards. The predicted MW of the recombinant 2His_6_‐ExsY is 19.4 kDa. Following Western blotting with anti‐His_6_ antibody, the full ladder of bands was clearer, extending from predicted monomer to hexamer. In contrast much of the crystal fraction did not enter the gel, although particularly on the Western blot, some hexamer and monomer could be detected. We found the yield of monomeric rExsY was only significantly increased when we incubated crystals at 95°C in the presence of 2 M DTT as well as urea and SDS; under these conditions, all crystallinity was lost (Supplementary Information Fig. S3). This is reminiscent of the behaviour of native exosporium.

### Electron crystallography

We analyzed exosporium and rExsY crystals by negative stain EM and image processing. All exosporium and rExsY fragments displayed unit cell parameters of *a* = *b* ≈ 80–85 Å, *γ* = 120°, similar to the ‘type II’ exosporium crystals described by us previously (Ball *et al*., 2008). Fourier phases for rExsY crystals and *Bt* 4D11 were consistent with hexagonal symmetry (Supplementary Information Tables S1 and S2). Projection maps are shown in Supplementary Information Fig. S4. The most prominent feature in all the projection maps is a lattice of rings, qualitatively similar to those we found previously (Ball *et al*., 2008; Terry *et al*., 2011). The projection map of crystals of rExsY (Supplementary Information Fig. S4C) was indistinguishable in gross staining pattern from the *Bt* 4D11 map (Supplementary Information Fig. S4B).

**Figure 7:**
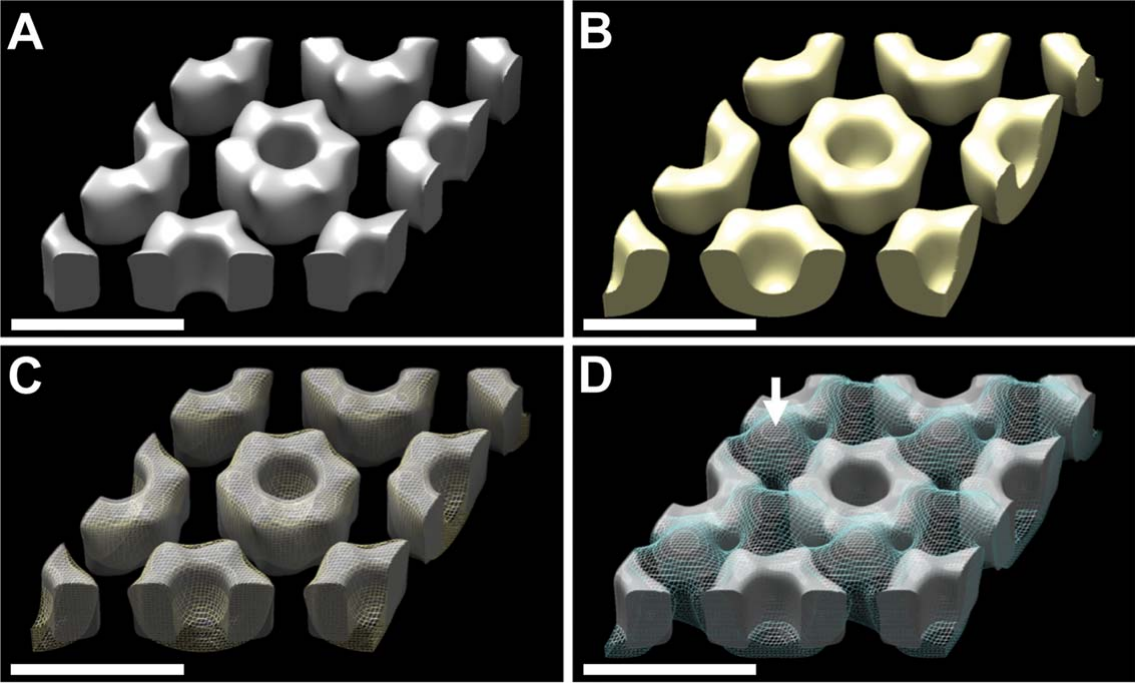
Surface comparisons between recombinant rExsY crystals and native exosporium crystals [Scale bars 8 nm]. A. rExsY crystal and (B) *B. thuringiensis* 4D11 whole spore exosporium. Overlays of (C) the rExsY crystal surface with *B. thuringiensis* 4D11 whole spore exosporium (yellow mesh) and (D) rExsY crystal surface with *B. cereus* 10876 exosporium (blue mesh). The arrow shows the ‘propeller’ motif linking ‘cups’ in *B. cereus*. The rExsY model was set at a threshold volume of ∼15 kDa/monomer. Surfaces rendered using UCSF CHIMERA (Pettersen *et al*., 2004).

For the reconstructions, interpolated Fourier amplitudes are shown in Supplementary Information Figs. S5 and S6. Figure 7A and B shows surface‐rendered views of the reconstructions; Fig. 7C shows a superimposition of these two density maps to give a best fit. The rExsY crystal is made up of an open lattice of hexameric ring‐like structures. The rings are ∼46 Å deep with central channels ∼27 Å in diameter. The *Bt* 4D11 exosporium is made up of a lattice of cup‐like structures, closed off at one end. The rExsY crystal has a near‐identical architecture except that there appears to be no ‘capping’ to form cups. Figure 7D shows a superimposition of the rExsY density with that of the exosporium of *B. cereus* 10876. The view is from the outside of the spore (Ball *et al*., 2008; Kailas *et al*, 2011) and shows in native exosporium an array of cup‐like structures connected by threefold‐symmetric linkers (arrow). Again, although the rExsY rings are superimposable on the native *B. cereus* exosporium, the latter has the additional embellishments of capping to form cups and in addition, the threefold‐symmetric linkers (Ball *et al*., 2008; Kailas *et al*, 2011).

## Discussion

### ExsY is an abundant and ubiquitous protein in exosporium

We have demonstrated that exosporium samples from *B. cereus* and *B. thuringiensis* 4D11 can be dissociated extensively to reveal the predominance of a relatively small number of intensely staining, sharp, low molecular weight bands (Fig. 3). This disruption of higher molecular weight complexes was reflected in the extensive dismantling of exosporium crystals (Fig. 2D).

The *Bt* 4D11 spores gave us an opportunity to examine the protein composition of an exosporium that maintains a crystalline basal layer but is free of hairy nap components. This exosporium was depleted in not only BclA, but its anchor protein, ExsFA (Fig. 4C). The most abundant exosporium protein (at 16 kDa) common to both *B. cereus* and *B. thuringiensis* strains is ExsY (Fig. 3), already implicated in exosporium assembly in both *B. cereus* and *B. anthracis* (Boydston *et al*., 2006; Johnson *et al*., 2006). It is possible that other proteins, which we did not interrogate by Western blotting, could also play a structural role. However, we conclude that ExsY is a major structural component of the exosporium basal layer (Fig. 3).

### ExsY defines the hexagonal crystalline framework of the exosporium

For all our rExsY constructs we found large insoluble crystalline sheets assembled within the *E. coli* host cell (Fig. 5). Remarkably, the distribution of density in the 2D projection map is virtually indistinguishable from that of the native exosporium crystals isolated from *Bt* 4D11 (Supplementary Information Fig. S4B and C). The 3D reconstructions from *Bt* 4D11 exosporium and rExsY show that the only significant difference between the two arrays is a closing off of the hexameric rings in the *Bt* 4D11 basal layer to form an array of ‘cups’ (Fig. 7A–C). Given this near‐identity in structure, we conclude that the major crystalline component within the exosporium of *Bt* 4D11 is ExsY but with additional components sealing off the ‘cups’.

The reconstructed density of rExsY (and that of *Bt* 4D11 exosporium) can also be superimposed on exosporium from *B. cereus* (Fig. 7D). The *B. cereus* exosporium has a structure nearly identical to that of *B. anthracis* (Ball *et al*., 2008). Thus we propose that ExsY forms the essential repeating unit defining most of the crystalline lattice of the exosporium basal layer, across the entire *B. cereus* group. Whilst a number of crystalline arrays have been identified in intact native spores (Holt and Leadbetter, 1969; Gould *et al*., 1970; Ebersold *et al*., 1981; Plomp *et al*., 2005a, 2005b), this is the first definitive demonstration of an *in vivo* crystalline array assembled from an identifiable spore protein.

In summary, Fig. 7A represents the structure of naked ExsY. The basic motif is one of a sixfold symmetric cylinder. In *B. thuringiensis* 4D11 (Fig. 7B) the cylinder is augmented by a tapered cap that closes it off at one end; this results in the cup‐like structure previously revealed by AFM (Kailas *et al*., 2011). In *B. cereus* 10876 (and *B. anthracis*) there is further augmentation with the prominent threefold symmetric ‘propeller’ motif that links the rings together (arrow in Fig. 7D).

### Additional protein links form at threefold symmetric positions

The ‘propeller’ motif (arrow in Fig. 7D) lies on the external face of the spore but the *Bt* 4D11 sample is missing this linker density (Fig. 7B and D). Variations in the ExsY amino acid sequence (95% identity) between *B. cereus* and *B. thuringiensis* HD1 (the parent strain of *Bt* 4D11) are too small to account for the extra volume of this linker. However, *Bt* 4D11 is devoid of ExsFA and BclA; this extra density seen in *B. cereus* could be accounted for by these extra protein components, given that most of the disordered BclA would be blurred out by our image processing. Indeed, we previously speculated that the trimeric BclA fibres may be anchored at the threefold symmetric bridges between the cups in the exosporium basal layer, via a link through a trimeric ExsFA anchor (Kailas *et al*., 2011). A 2D projection analysis of wild type and *exsFA* (*bxpB*) mutant strains of the closely related *B. anthracis* exosporium is entirely consistent with this interpretation. This study localized the putative anchor point to somewhere on the cup rim (Rodenburg *et al*., 2014), but in our 3D study we have now determined a precise location. Thus, we propose that the base of the putative ExsFA/BclA complex is located at the position of threefold symmetry at the centre of the ‘propeller’; it is now seen to link the ExsY rings together. Notably, ExsFA, BclA and ExsY have been observed to co‐migrate in SDS‐PAGE in positions corresponding to higher order complexes (Redmond *et al*., 2004). Taken together with our interpretation of the threefold linkers of ExsFA and BclA bridging ExsY oligomers, this does suggest that these three proteins interact in a single complex (Todd *et al*., 2003; Redmond *et al*., 2004; Steichen *et al*., 2005). However, the stability of the pure rExsY crystals demonstrates that this ‘propeller motif’ linker is not an absolute requirement to hold together the ExsY rings of the basal layer. Consistent with this, while ExsFA and ExsFB are important for conferring exosporium stability on the spore, they do not appear essential in maintaining basal layer crystallinity in sloughed‐off fragments from *exsF* spores (Sylvestre *et al*., 2005).

### Exosporium is stabilized through disulphide bonding of ExsY

A high cysteine content is characteristic of ExsY and its homologues and a high proportion of the cysteine residues are in conserved positions throughout this family. We now have strong evidence that the cysteines' role includes cross‐linking (Zhang *et al*., 1993; Jiang *et al*., 2015). Cross‐linking may occur at three levels: within single polypeptide chains of ExsY; between ExsY subunits within individual hexamers and between hexamers, thus cross‐linking the entire lattice. Our analyses suggest the presence of both inter‐chain disulphides within hexamers and also between hexamers in the lattice. Figure 6B shows that both heat and reducing agent are required for the complete loss of detectable hexamer. We previously found analogous behaviour for CotY_Bs_ hexamers and showed that higher order assemblies also required reducing agent for disassembly (Jiang *et al*., 2015). Treatment of rExsY crystals with a combination of both heat and DTT was required to destroy any ordered crystallinity. Some monomeric material was extractable upon 2M DTT treatment alone (Fig. 6, lane 3), however we found essentially the same population density of large crystals as for untreated samples.

### On the role of self‐organization and cross‐linking in exosporium assembly

rExsY assembles into crystalline sheets in the cytoplasm of the *E. coli* host. This remarkable behaviour suggests that ExsY is capable of self‐organization; it is unlikely that *E. coli* would have specific machinery for the assembly of this recombinant protein. We have previously reported on a propensity for self‐assembly in a number of spore coat proteins of *B. subtilis* (which does not have an exosporium), including the ExsY orthologue, CotY_Bs_. How does this self‐organization incorporate disulphide cross‐linking in the normally reducing environment of the host cell and by inference how could such cross‐links be formed in the native sporangium? The hexameric nature of ExsY combined with the crystal packing over and above this may play a role; the formation of multiple disulphide bonds as ExsY subunits are recruited and precisely positioned into the highly symmetric growing hexamers and into the higher order lattice could be a highly cooperative process; such cooperativity could form an exquisite mechanism for ensuring that these disulphides are exceptionally strong and resistant to the normally reducing intracellular environment (Jiang *et al*., 2015). Cooperative disulphide bond formation has been demonstrated in protein folding experiments, where the equilibrium constant for additional disulphides is very substantially increased over that of a single bond (Chau and Nelson, 1992). In an analogous effect, in ExsY crystals, the positioning and locking of subunits into the growing lattice could enhance the local sulphydryl concentration thus driving the formation of additional cross‐links.

Exosporium assembly starts at a ‘cap’ on one pole of the forespore (Ohye and Murrell, 1973). In the absence of ExsY, assembly terminates after cap formation (Boydston *et al*., 2006; Johnson *et al*., 2006). This suggests that the remaining two thirds of the exosporium is predominantly made of ExsY (Boydston *et al*., 2006; Steichen *et al*., 2007). ExsY self‐assembly is likely to help drive the formation of the basal‐layer template. CotY is preferentially located in the cap, to some degree controlled by exclusion involving a secondary protein with a collagen‐like region, BclB (Thompson *et al*., 2012). CotY alone may be sufficient for cap self‐assembly (Boydston *et al*., 2006; Steichen *et al*., 2007; Thompson *et al*, 2012), but not for completing the assembly beyond. Recombinant CotY can itself organize into crystalline sheets (Supplementary Information Fig. S2), suggesting that self‐organization is also an essential feature in cap assembly. We do not know how cap growth is limited but our observation that recombinant CotY forms crystals that are more disordered than those of rExsY suggests that defects may be more prevalent in the cap. Ultimately, ExsY may dominate crystal growth around the rest of the spore, favoured kinetically and thermodynamically over CotY.

**Figure 8:**
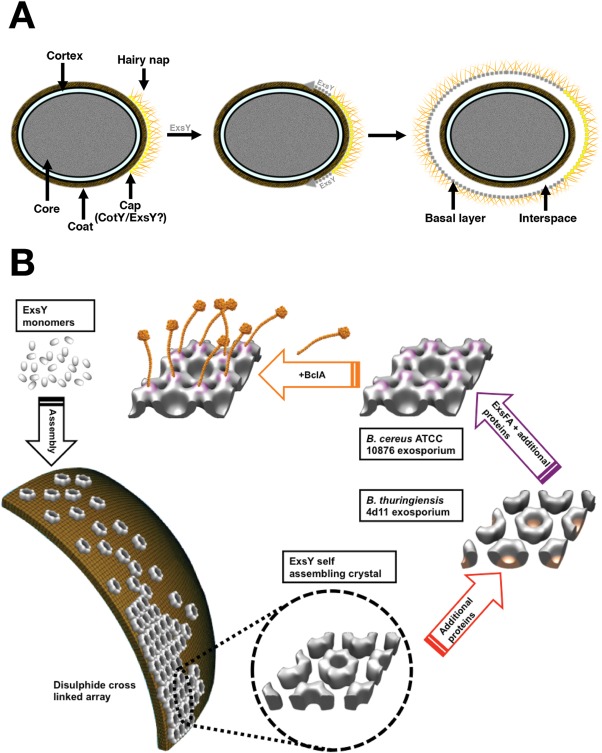
Schematic diagram of the hierarchy of assembly states of exosporium in the *B. cereus* group. A. Main stages in the assembly based on the outline by Henriques and Moran (2007). The greater part of the exosporium extends out from the initial cap through assembly of ExsY into the basal layer. Note that the relative timing of hairy nap formation at the cap and ExsY extension is not certain. B. Monomeric ExsY self‐assembles into a hexameric disulphide cross‐linked array. In the native *B. thuringiensis* 4D11 exosporium additional material rounds off the ‘cup’. The *B. cereus* 10876 structure indicates that ExsFA must be added to form an anchor for the BclA filaments of the hairy nap.

In the sporangium, a thin ‘sac’ is proposed to surround the spore (Boydston *et al*., 2006; Henriques and Moran, 2007) and may define a preferred interface for ExsY assembly. Figure 8 shows a model for the role of ExsY in exosporium growth (Henriques and Moran, 2007; Steichen *et al*., 2007). Assembly starts by formation of the sac sublayer (Fig. 8A) followed by cap formation. The cap may incorporate domains of self‐assembled CotY and ExsY. For assembly to start here may require anchoring proteins e.g. ExsA and CotE (Bailey‐Smith *et al*., 2005; Giorno *et al*., 2007; Henriques and Moran, 2007). The basal layer extends from the ‘cap’ around the developing spore through self‐organization of predominantly ExsY; this is summarized in Fig. 8B although this figure does not necessarily imply a strict temporal sequence, rather a hierarchy of assembly states. The main features are as follows:

- ExsY forms hexamers.
- ExsY is targeted to the ‘sac’ and assembles into a two‐dimensional lattice (Henriques and Moran, 2007). Lattice growth may be initiated at the ‘cap’ periphery.
- Disulphide cross‐links are formed (Fig. 6).
- Additional proteins account for the plug of density ‘closing’ off one end of each ring leading to the characteristic ‘cup’ structures (Fig. 7).
- ExsFA/BxpB and BclA are coexpressed and assemble together on the developing exosporium.

### Functional implications

The capacity for ExsY and CotY to self‐organize into an ordered lattice of high symmetry gives a possible elegant solution to growing the exosporium and the problem of establishing disulphide cross‐links within the intra‐cellular environment. The result is a highly stable assembly. Despite this, the exosporium is flexible enough to form tight folds which may confer advantages in adhesion to surfaces (Kailas *et al*., 2011). It is possible that cross‐linking is not fully saturated in the natural exosporium‐ indeed it is evident that a proportion of the natural exosporium can be reversibly disassembled without reducing agents (Fig. 2C).

The critical structural role played by ExsY is evident from an *exsY* strain of *B. cereus* which makes only a small terminal cap and an *exsYcotY* strain which is completely devoid of exosporium. Conversely a *cotY* strain is able to make an apparently intact exosporium (Boydston *et al*., 2006; Johnson *et al*., 2006; Steichen *et al*., 2007); our model would predict that the exosporium basal layer in this case is made entirely through ExsY self‐assembly.

The underlying principles governing the assembly of the exosporium basal layer may be similar to those across all the distantly related *Bacillus* and *Clostridium* species. Highly ordered self‐assembly of protein components into robust two dimensional arrays through disulphide cross‐linking has been demonstrated by CotY_Bs_ in *B. subtilis* spore coats (Jiang *et al*., 2015). This may emerge as a general strategy to aid in the construction of highly resistant cellular structures across other species. For example it is notable that a cysteine‐rich protein, CdeC is required for ‘exosporium’ morphogenesis and coat assembly in *C. difficile* spores (Barra‐Carrasco *et al*., 2013). Moreover, we have recently demonstrated hexagonal packing in the exosporium in a number of other Clostridium spp. and also identified a number of cysteine‐rich exosporium proteins, CsxA, CsxB and CsxC; these bear no sequence homology to ExsY (Janganan *et al*., 2016).

## Experimental procedures

Additional experimental information is provided in Supplementary Methods in Supplementary Information.

### Endospore preparation

Vegetative cells were grown in nutrient broth and spores prepared using CCY media as previously described (Todd *et al*., 2003).

### Isolation of exosporium

Exosporium fragments were isolated using the French press method (‘unwashed’) and washed with salt and detergent buffers (‘fully washed’) as described by Terry *et al*. (Terry *et al*., 2011).

### SDS‐PAGE and Western blotting

Exosporium proteins were resuspended in solubilization buffer (50 mM CHES pH 9.8, 8 M urea, 2% SDS, 200 mM DTT); they were incubated at 90°C for 20 min to disrupt exosporium complexes for separation by SDS‐PAGE and Western blotting as previously described (Terry *et al*., 2011), using 10% NuPAGE 1 mm Bis‐Tris pre‐cast gels (Invitrogen).

### Disassembly of the exosporium

SDS (1%) or urea (8 M) were added to 0.1 mg of fully washed exosporium fragments and incubated at 37°C for 1 h. To remove insoluble material, the sample was centrifuged at 10,000 x g for 15 min. The solubilized fraction was dialysed against 1 l of deionized water containing 0.05% (w/v) of sodium azide (Fluka) using 3500 MWCO Slide‐A‐Lyzer Mini Dialysis Units (Pierce). Fully disrupted exosporium samples were resuspended in 8 M urea, 2% (w/v) SDS with or without the addition of 0.2 M DTT and heated to 90°C for 20 min. Samples were dialysed using 3500 MWCO Slide‐A‐Lyzer Mini Dialysis Units.

### Expression and purification of ExsY and CotY

The *exsY* gene from *B. cereus* ATCC 10876 was cloned into pET28a, generating a construct with both N‐ and C‐terminal poly‐histidine tags (2His_6_‐ExsY). An untagged *exsY* construct was produced by cloning into pCOLADuet‐1. ExsY over‐expression was carried out in *E. coli*. Crystals of his‐tagged ExsY were collected by batch purification using NiNTA Agarose beads. C‐terminal poly‐histidine tagged CotY crystals were similarly isolated.

### Disassembly of recombinant ExsY crystals

As described previously for *B. subtilis* CotY (Jiang *et al*., 2015), except that 95°C was used.

### Electron microscopy

For details, see Supplementary Information. Samples were examined on a Phillips CM100 at 100 kV. Images were collected on a 1K x 1K Gatan CCD camera. A total of 51 images of ExsY crystals and 70 images of *B. thuringiensis* 4D11 exosporium, were collected for processing. The specimen tilt angle ranged from −50° to +50°.

### Image processing and 3D reconstruction

Images were initially processed using the *2dx* suite (Gipson *et al*., 2007). Output Fourier phases and amplitude data were subsequently merged using the MRC program ORIGTILTK followed by 3D map generation using the CCP4 suite of programs (Collaborative Computational Project, 1994; Crowther *et al*., 1996).

## Author contributions

PAB, AM, CT and SJ designed research. CT, SJ, DSR, QW and ST performed research. CT, SJ, PAB and AM analyzed data. PAB, AM, CT and SJ wrote the paper.

## Supporting information

Supporting Information
