# Supplementary material for: Molecular tiling on the surface of a bacterial spore – the exosporium of the Bacillus anthracis/cereus/thuringiensis group
Source: Mol Microbiol. 2017 Mar 8;104(4):539–52. doi: 10.1111/mmi.13650 (PMC5434927; doi:10.1111/mmi.13650)
Supplement: Supplementary file 1 — Supporting Information [file MMI-104-539-s001.pdf]

Molecular tiling on the surface of a bacterial spore- the exosporium of the *Bacillus anthracis/cereus/thuringiensis* group

Cassandra Terry,[+] Shuo Jiang,[+] David S. Radford, Qiang Wan, Svetomir Tzokov, Anne Moir, Per A. Bullough[#]

Krebs Institute for Biomolecular Research, Department of Molecular Biology and Biotechnology, University of Sheffield, Sheffield, United Kingdom

# Correspondence and requests for materials should be addressed to P.A.B.

(email : p.bullough@sheffield.ac.uk)

[+] These authors contributed equally to this work.

Supplementary Fig. 1. A proportion of exosporium crystal fragments are solubilised and broken into small spheroidal particles after SDS treatment.

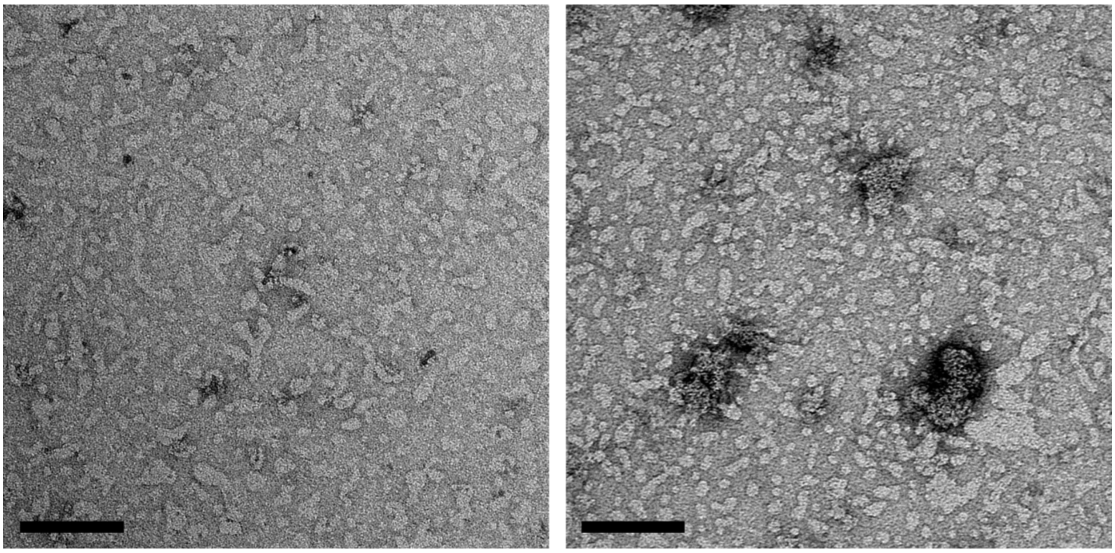

Small ‘spheroidal particles’ 10 – 45 nm in dimension plus some small crystalline fragments that do not pellet during centrifugation are observed in the soluble fraction after incubation of *B. cereus* 10876 exosporium crystals with 1% SDS and analysis by negative stain electron microscopy. Scale bar, 100 nm.

Supplementary Fig. 2. Electron micrograph and computed Fourier transform (inset) of heterologously expressed *B. cereus* CotY crystal extracted from *E. coli*.

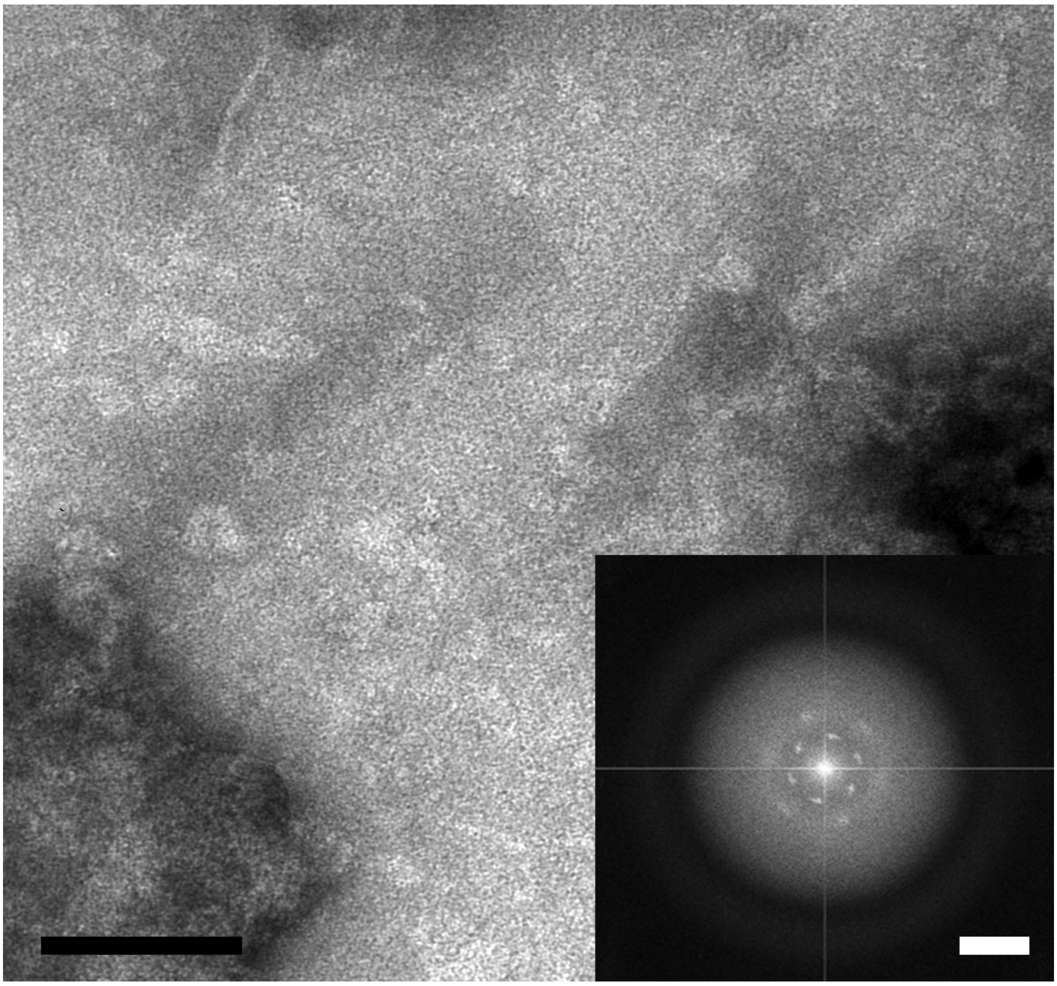

Crystals with well-defined diffraction spots were rare in comparison to heterologously overexpression ExsY. Unit cell parameters for CotY showed *a* = *b* = ~ 80 Å, © ~ 120°. (Scale bars, 100 nm in micrograph and 0.3 nm^{-1} inset).

Supplementary Fig. 3. Treatment of 2His$_6$-ExsY with urea, SDS, DTT and heating yielded large non-crystalline aggregates and small globular particles.

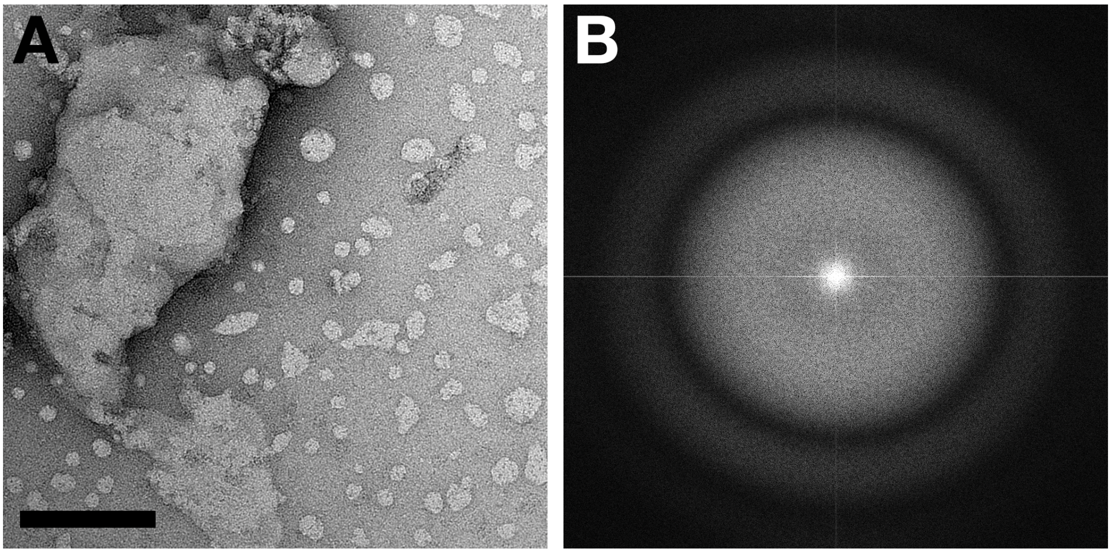

(A) Large amorphous aggregates and small globular particles were found after treatment with 8M urea, 2% SDS, 2 M DTT and 95° C heating for 20 minutes. (B) Computer Fourier transform of (A) indicates the loss of an ordered lattice. Scale bar, 100 nm.

Supplementary Table 1. The internal phase residuals determined after the imposition of all allowed two-sided plane groups calculated from one of the micrographs of ExsY crystals.

Two sided plane group	Phase residual (°) (90° random)	Number of Comparisons	Target residual based on statistics taking Friedel weight into account (°)
*p*1	14.0	48	
*p*2	21.2	24	20.0
*p*3	8.2*	48	14.0
*p*312	36.2	102	14.4
*p*321	27.3	105	14.5
*p*6	14.4*	120	15.2
p622	38.9	231	14.6

Internal phase residuals were determined from spots of IQ1-1Q5 to 20 Å resolution (Crowther *et al.,* 1996, Valpuesta *et al.,* 1994). The values marked with * are acceptable candidates for the symmetry as the experimental phase residual is better than that expected, based on the signal-to-noise ratio.

Supplementary Table 2. The internal phase residuals determined after the imposition of all allowed two-sided plane groups calculated from one of the micrographs of *B. thuringiensis* 4D11 exosporium crystals.

Two sided plane group	Phase residual (°) (90° random)	Number of Comparisons	Target residual based on statistics taking Friedel weight into account (°)
*p*1	23.1	32	
*p*2	25.5*	16	33.6
*p*3	7.6*	30	23.1
*p*312	14.4*	60	24.2
*p*321	8.1*	61	24.3
*p*6	17.2*	32	25.3
p622	16.4*	137	24.4

Internal phase residuals were determined from spots of IQ1-1Q5 to 20 Å resolution (Crowther *et al.,* 1996, Valpuesta *et al.,* 1994). The values marked with * are acceptable candidates for the symmetry as the experimental phase residual is better than that expected, based on the signal-to-noise ratio.

Supplementary Fig. 4. Projection maps of *B. cereus* ATCC 10876 exosporium, *B. thuringiensis* 4D11 exosporium and ExsY self-assembling crystal.

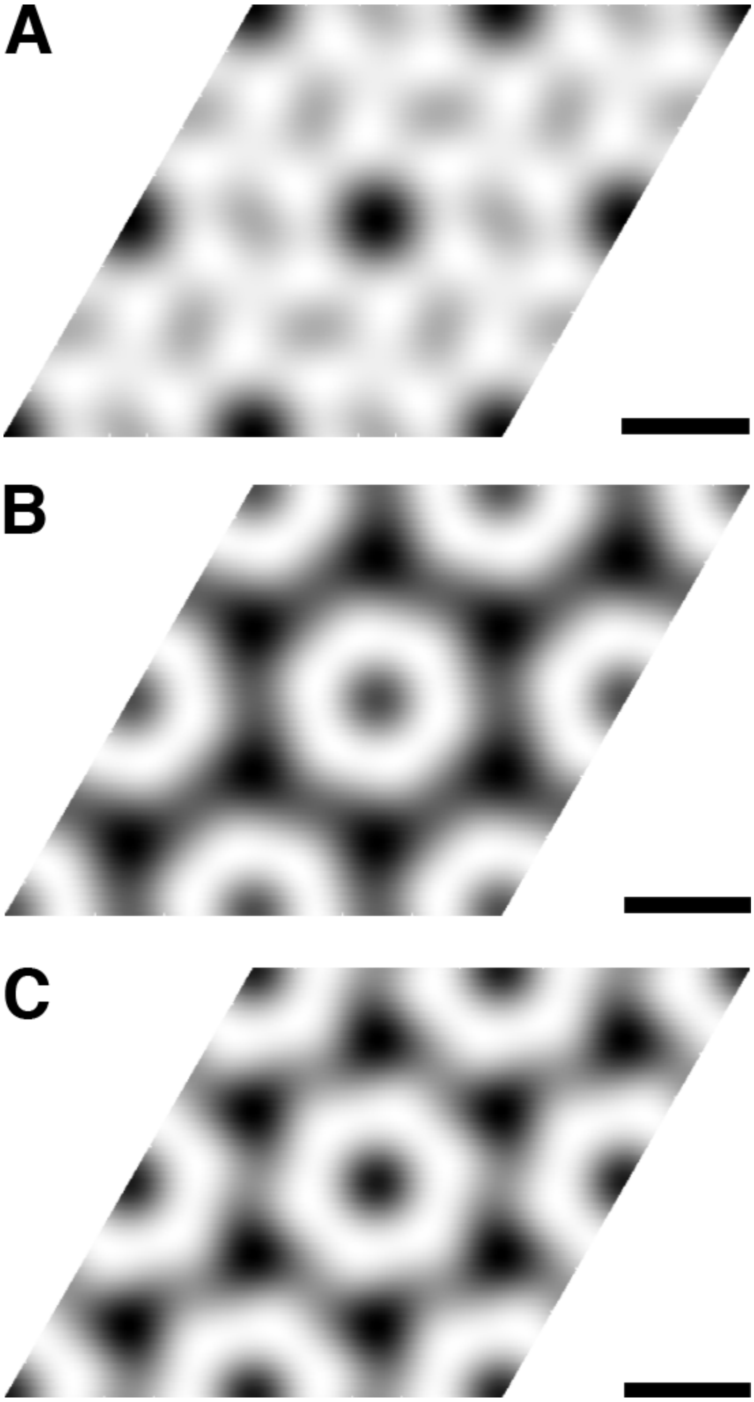

Averaged greyscale projection map of negatively stained (A) *B. cereus* ATCC 10876 exosporium, (B) *B. thuringiensis* 4D11 exosporium and (C) ExsY self-assembled crystal. All projection maps have sixfold (*p*6) symmetry enforced. Light regions indicate stain-excluding i.e. proteinaceous regions. Scale bars, ~40 Å.

Supplementary Fig. 5. Amplitude and phase variation along z* for selected *h*, *k* values for ExsY crystals. *p*6 symmetry was imposed.

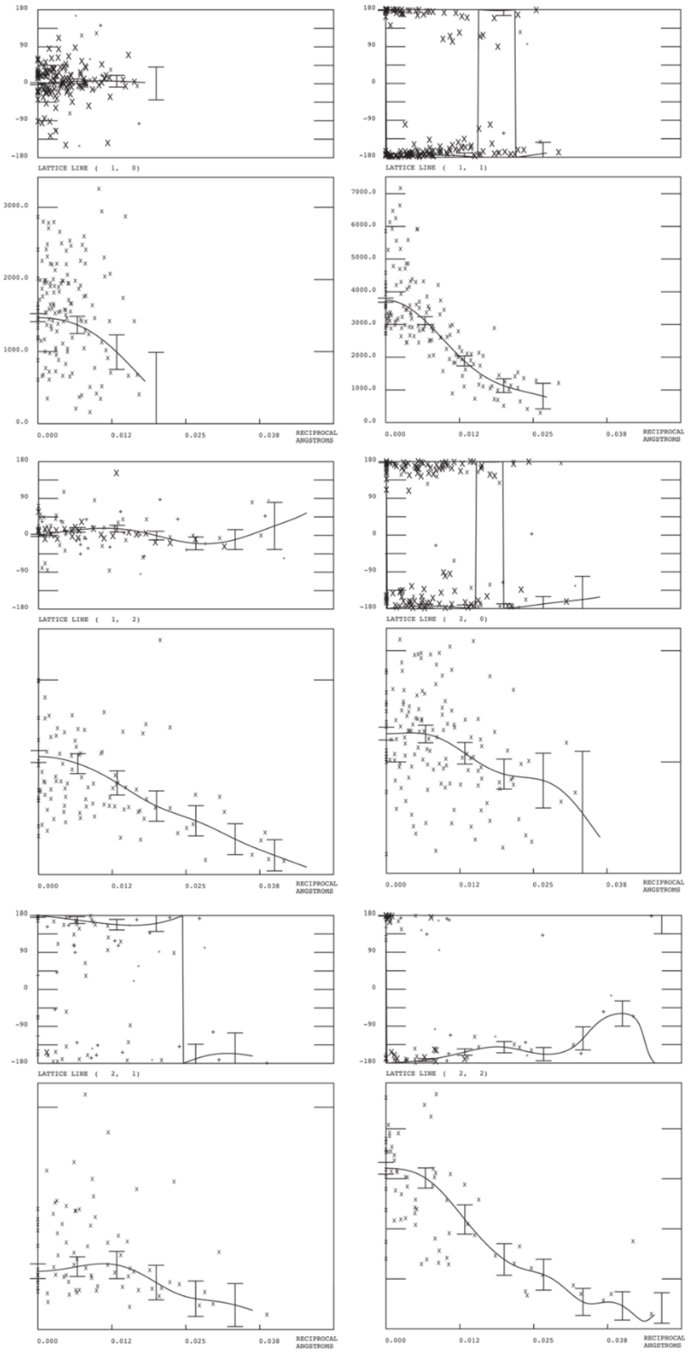

Lattice lines showing the phase variation along the z* axis in ° (top panels) and amplitude variation in arbitrary units (lower panels). Horizontal axis displays reciprocal distance from the origin of the lattice line. Standard error of fitted amplitude and phase values is represented in the error bars.

Supplementary Fig. 6. Amplitude and phase variation along z* for selected *h*, *k* values for *B. thuringiensis* 4D11 exosporium crystals. *p*6 symmetry was imposed.

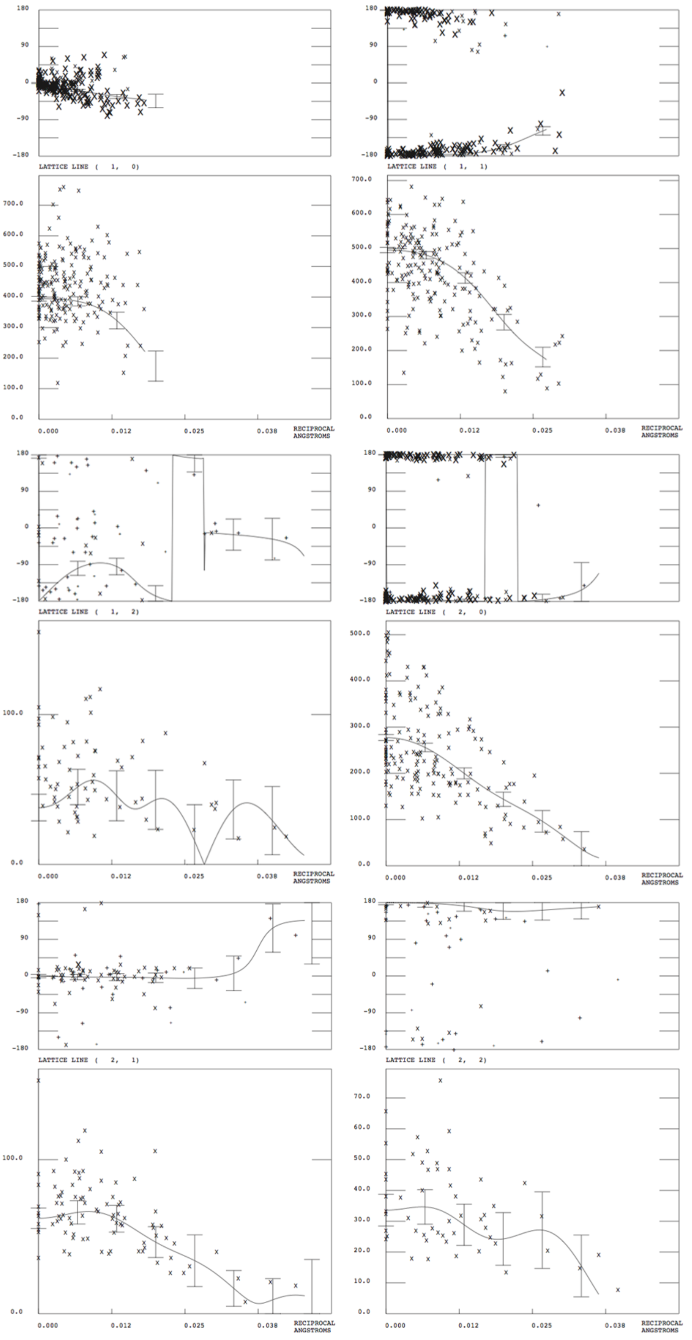

Lattice lines showing the phase variation along the z* axis in ° (top panels) and amplitude variation in arbitrary units (lower panels). Horizontal axis displays reciprocal distance from the origin of the lattice line. Standard error of fitted amplitude and phase values is represented in the error bars.

Supplementary Methods

Endospore preparation. Vegetative cells were grown in nutrient broth and spores prepared using CCY media as previously described (Todd *et al*., 2003, Stewart *et al*., 1981). Spores were harvested when the culture contained >95% free spores followed by 10 washes in sterile ice-cold water to remove debris and vegetative cells. Washed spore pellets were resuspended in 50 mM Tris-HCl, 0.5 mM EDTA pH 7.5 at 20-50 mg ml^{-1} dry weight, and stored at -20 °C.

Isolation of exosporium fragments. Exosporium fragments were isolated using the French press method ('unwashed') and washed with salt and detergent buffers ('fully washed') as described by Terry *et al.,* (2011). The unwashed exosporium was first washed in TEP buffer (50 mM Tris-HCl pH 7.2, 10 mM EDTA, 1 mM PMSF) containing 0.5 M KCl and 1% (v/v) glycerol. 1 M NaCl was used in the second wash followed by TEP buffer containing 0.1% (w/v) SDS. TEP buffer was used as the final wash. The exosporium was recovered after each wash step by ultracentrifugation at 145,000 x g for 2 hours. *B. thuringiensis* 4D11 exosporium used for image processing was isolated from spores grown with gentle agitation and sonicated to remove the exosporium. Protein concentration was determined using a BCA™ Protein Assay Kit (Pierce).

SDS-PAGE and western blotting. Exosporium proteins were resuspended in solubilisation buffer (50 mM CHES pH 9.8, 8 M urea, 2% SDS, 200 mM DTT) and incubated at 90 °C for 20 minutes to disrupt exosporium complexes for separation by SDS-PAGE and western blotting as previously described (Terry *et al*., 2011) using 10% NuPAGE 1 mm Bis-Tris pre-cast gels (Invitrogen).

Mouse monoclonal anti-BclA antibodies (Terry *et al*., 2011) were used at a concentration of 2 μg ml^{-1}. Rabbit polyclonal anti-CotE antibodies (Giorno *et al*., 2007) were used at a

concentration of 5 µg ml^{-1}. Rabbit polyclonal anti-ExsK antibodies (Severson *et al.*, 2009) were used at a final concentration of 6 µg ml^{-1}. Polyclonal anti-ExsFA antibodies (Sylvestre *et al.*, 2005) were used at a concentration of 1/2000. We used as negative controls either mouse anti-human B cell CD23 IgG primary antibodies (Dako) or anti-Goat IgG whole molecule produced in rabbit (Sigma-Aldrich). Polyclonal antibody preparations were cleaned using an Immobilized *E. coli* Lysate Kit (Thermo Scientific) to remove any contaminating anti-*E. coli* antibodies.

Expression and purification of ExsY crystals. One Shot® TOP10 Electrocomp™ *E. coli* (Invitrogen™) was used to maintain all plasmids used in this study. The *exsY* gene from *B. cereus* ATCC 10876, with the last two amino acids deleted (V153 and K154) to replicate the *exsY* gene from *B. anthracis*, was cloned into *Nde*I- and *Xho*I- digested pET28a, generating both N- and C-terminal poly-histidine tags and a construct with tags at both termini ($2His_6$-ExsY). An untagged *exsY* construct was produced by cloning into NdeI-XhoI digested pCOLADuet-1 vector. ExsY protein over-expression was carried out in *E. coli* strain BL21(DE3)pLysS and grown in LB media containing the appropriate antibiotic marker. Cells were grown at 37 °C with vigorous shaking until an OD_{600} of 0.6 before inducing with 1 mM IPTG for 4 hours and collected by centrifugation. Cells were resuspended in urea buffer (8 M urea, 150 mM NaCl, 25 mM Tris-HCl, pH 8.0) and disrupted by sonication. Crystals of his-tagged ExsY were collected by batch purification using NiNTA Agarose beads (Qiagen) and eluted using 300 mM imidazole in urea buffer. Crystals were isolated by ultracentrifugation of eluate at 39,000 x g and resuspended in urea buffer.

Electron microscopy. 5 µl of spores at a concentration of ~ 4 mg ml^{-1} and 3 µl of exosporium at ~ 0.6 mg ml^{-1} were loaded onto carbon coated grids and stained with 0.75 % uranyl formate as previously described (Ball *et al.*, 2008). Samples were examined on a Phillips CM100 transmission electron microscope at an accelerating voltage of 100 kV. Digital images were collected on a 1K x 1K Gatan Multiscan 794 CCD camera. Fragments of

exosporium or 2D protein crystals were examined as above. Electron micrographs were recorded at a magnification of ~52,000 X and at ~400 nm underfocus. A total of 51 images of ExsY crystals and 70 images of *B. thuringiensis* 4D11 exosporium, were collected for processing. The specimen tilt ranged from -50° to +50°.

Supplemental references

Ball, D.A., Taylor, R., Todd, S.J., Redmond, C., Couture-Tosi, E., Sylvestre P, *et al*. (2008). Structure of the exosporium and sublayers of spores of the *Bacillus cereus* family revealed by electron crystallography. *Mol Microbiol* **68**: 947–958.

Crowther, R.A., Henderson, R., Smith, J.M.**,** (1996). MRC image processing programs. *J Struct Biol* **116**: 9-16.

Giorno, R., Bozue, J., Cote, C., Wenzel, T., Moody, K.S., Mallozzi, M., *et al*. (2007). Morphogenesis of the *Bacillus anthracis* spore. *J Bacteriol* **189**: 691-705.

Severson, K.M., Mallozzi, M., Bozue, J., Welkos, S.L., Cote, C.K., Knight, K.L., *et al*. (2009). Roles of the *Bacillus anthracis* spore protein ExsK in exosporium maturation and germination. *J Bacteriol* **191**: 7587–7596.

Stewart, G.S., Johnstone, K., Hagelberg, E., Ellar, D.J., (1981). Commitment of bacterial spores to germinate. A measure of the trigger reaction. *Biochem J* **198**:101–106.54.

Sylvestre, P., Couture-Tosi, E., Mock, M., (2005). Contribution of ExsFA and ExsFB proteins to the localization of BclA on the spore surface and to the stability of the *Bacillus anthracis* exosporium. *J Bacteriol* **187**: 5122–5128.

Terry, C., Shepherd, A., Radford, D.S., Moir, A., Bullough, P.A., (2011). YwdL in *Bacillus cereus*: its role in germination and exosporium structure. *PLoS ONE* **6**: e23801.

Todd, S.J., Moir, A.J.G., Johnson, M.J., Moir, A., (2003). Genes of *Bacillus cereus* and *Bacillus anthracis* encoding proteins of the exosporium. *J Bacteriol* **185**: 3373–3378.

Valpuesta, J.M., Carrascosa, J.L., Henderson, R., (1994). Analysis of electron microscope images and electron diffraction patterns of thin crystals of phi 29 connectors in ice. *J Mol Biol* **240**: 281-7.
